# Extensive antagonistic genetic underpinnings of sex disparities in Alzheimer’s disease and hypertension

**DOI:** 10.1002/alz.091324

**Published:** 2025-01-03

**Authors:** Alexander M. Kulminski, Fan Feng, Irina Kulminskaya, Alireza Nazarian, Elena Loiko, Yury Loika

**Affiliations:** ^1^ Social Science Research Institute, Duke University, Durham, NC USA

## Abstract

**Background:**

The Alzheimer’s Association reports that more than two‐thirds of the approximately 5 million Alzheimer’s disease (AD) cases in the USA are women. With studies suggesting a high genetic heritability for AD, gaining a better understanding of the genetic architecture underlying sex disparities in AD and its risk factors could enhance the understanding of their mechanisms.

**Method:**

We conducted genome‐wide association studies (GWAS) and pleiotropic meta‐analysis of AD and its risk factor—hypertension—in men and women of European ancestry. The AD GWAS focused on 168,962 men (including 6,229 cases) and 199,668 women (including 7,628 cases) from 10 cohorts. The hypertension GWAS included 177,597 men (96,835 cases) and 205,482 women (88,861 cases) from eight of the 10 AD cohorts. Sex‐specific associations were characterized by the Wald chi‐square test and Fisher’s method, and their significance was assessed at a false discovery rate *q*‐value of 5% or less.

**Result:**

GWAS of AD and hypertension identified 176 and 195 loci, respectively, on all chromosomes with the differences in the effects between sexes attaining *q*<0.05. Associations with AD were of the same directions in men and women only in one—the *APOE* gene—locus, with significantly larger adverse effect for the ε4‐encoding rs429358 polymorphism in women (β = 1.35, CI = 1.30‐1.41) than men (β = 1.16, CI = 1.10‐1.22). Associations with hypertension were of the same directions in each sex in 74 of 195 loci on all chromosomes, except chromosome 14. The ε4 allele showed no association with hypertension in either sex. No significant differences between sexes in the significant protective associations with AD and hypertension were identified for the ε2 allele. Our analyses revealed seven pleiotropic loci mapped to *COL24A1, MGC4859‐PHF14, MFHAS1‐ERI1, SBF2, KICS2, BCL11B*, and *RPAP1* gene clusters characterized by the differences in the associations with AD and hypertension between sexes at *q*<0.05. In three underlined clusters, the associations with hypertension were of the same direction in men and women.

**Conclusion:**

Although the *APOE* ε4 allele partly underlies AD‐related sex disparities, the most substantial contribution comes from antagonistic relationships between genes and AD in men and women. Sex‐specific pleiotropic loci suggest contribution of hypertension to these AD‐related sex disparities.

